# The Burden of Non-Communicable Disease in Transition Communities in an Asian Megacity: Baseline Findings from a Cohort Study in Karachi, Pakistan

**DOI:** 10.1371/journal.pone.0056008

**Published:** 2013-02-13

**Authors:** Faisal S. Khan, Ismat Lotia-Farrukh, Aamir J. Khan, Saad Tariq Siddiqui, Sana Zehra Sajun, Amyn Abdul Malik, Aziza Burfat, Mohammad Hussham Arshad, Andrew J. Codlin, Belinda M. Reininger, Joseph B. McCormick, Nadeem Afridi, Susan P. Fisher-Hoch

**Affiliations:** 1 Indus Hospital Research Center, Korangi Crossing, Karachi, Pakistan; 2 Interactive Research & Development, Shahrah-e-Faisal, Karachi, Pakistan; 3 University of Texas School of Public Health, Brownsville, Texas, United States of America; 4 Association of Pakistani-Descent Cardiologists of North America, Wellington, Florida, United States of America; Fundación para la Prevención y el Control de las Enfermedades Crónicas No Transmisibles en América Latina (FunPRECAL), Argentina

## Abstract

**Background:**

The demographic transition in South Asia coupled with unplanned urbanization and lifestyle changes are increasing the burden of non-communicable disease (NCD) where infectious diseases are still highly prevalent. The true magnitude and impact of this double burden of disease, although predicted to be immense, is largely unknown due to the absence of recent, population-based longitudinal data. The present study was designed as a unique ‘Framingham-like’ Pakistan cohort with the objective of measuring the prevalence and risk factors for hypertension, obesity, diabetes, coronary artery disease and hepatitis B and C infection in a multi-ethnic, middle to low income population of Karachi, Pakistan.

**Methods:**

We selected two administrative areas from a private charitable hospital’s catchment population for enrolment of a random selection of cohort households in Karachi, Pakistan. A baseline survey measured the prevalence and risk factors for hypertension, obesity, diabetes, coronary artery disease and hepatitis B and C infection.

**Results:**

Six hundred and sixty-seven households were enrolled between March 2010 and August 2011. A majority of households lived in permanent structures (85%) with access to basic utilities (77%) and sanitation facilities (98%) but limited access to clean drinking water (68%). Households had high ownership of communication technologies in the form of cable television (69%) and mobile phones (83%). Risk factors for NCD, such as tobacco use (45%), overweight (20%), abdominal obesity (53%), hypertension (18%), diabetes (8%) and pre-diabetes (40%) were high. At the same time, infectious diseases such as hepatitis B (24%) and hepatitis C (8%) were prevalent in this population.

**Conclusion:**

Our findings highlight the need to monitor risk factors and disease trends through longitudinal research in high-burden transition communities in the context of rapid urbanization and changing lifestyles. They also demonstrate the urgency of public health intervention programs tailored for these transition communities.

## Introduction

The developing world is in the midst of a profound health transition. In many developing communities the persistent burden of infectious diseases is now coupled with the rising tide of non-communicable disease (NCD). Rapid urbanization and increasing globalization in low and middle-income countries (LMIC) has led to changes in lifestyles, food availability, and tobacco consumption in populations that continue to experience high morbidity and mortality from communicable diseases [1]. NCDs, defined as diseases of long duration and generally slow progression, are now the leading global cause of death [2]. LMICs currently account for 80% of all NCD related mortality with totals considerably exceeding those due to communicable diseases, maternal, neonatal and injury related deaths combined [1]. The World Economic Forum estimates that by 2030 the global cost of NCDs will reach $47 trillion dollars, confirming that their threat to both global health and the global economy is among our greatest twenty-first century challenges [2].

Annual NCD deaths are projected to continue to rise worldwide with the greatest increase expected to be seen in LMICs in Africa, South-East Asia and the Eastern Mediterranean, where they will increase by over 20% [1]. The rise in NCDs largely stems from four behavioral risk factors: tobacco use, unhealthy diet, insufficient physical activity and the harmful use of alcohol [1]. Nearly 80% of the world’s one billion smokers live in LMICs that account for 3.4 million deaths, and these are projected to double by 2030 [3]. Globalization and urbanization are making processed foods - high in refined starch, sugar, salt and unhealthy fats - cheaply available to consumers and encouraging sedentary lifestyles in these regions [4]. As a result overweight and obesity are on the rise in the developing world [5]–[7].

Behavioral risk factors common to NCD are characteristic of rapid urbanization, economic transition and 21^st^ century lifestyles emerging in Asian megacities [8]. More than 60% of the increase in the world’s urban population over the next three decades will occur in Asia, where it will exceed 2.6 billion by 2030, and where 16 of the world’s 24 megacities are found [9]. The prevalence of diabetes has risen more rapidly in South Asia than in other regions of the globe, and cardiovascular risk is already the highest in the world [10], [11]. Pakistan has the highest rate of urbanization in South Asia and a population of 187 million with an annual per-capita health expenditure equivalent to US$63.90. In terms of the number of lives lost due to ill-health, disability, and early death (DALYs), NCDs account for 59% of the total disease burden in Pakistan with cardiovascular disease representing the largest share followed by chronic respiratory diseases, cancer and diabetes [11]–[13]. In the past decade, weak economic growth and poor infrastructure development in rural areas, unrelenting population growth rates, and internal security challenges have displaced large numbers from rural communities, resulting in an influx into urban centers, the largest of which is Karachi [13], [14]. Located on the Arabian Sea next to the Indus River delta, Karachi is the country’s economic hub with an estimated 2011 population of 21.7 million representing all major ethnic groups [15]. Against this background, Karachi offers a unique opportunity to study and measure more precisely the burden of non-communicable diseases and their associated risk factors in a South Asian megacity.

Longitudinal studies in randomly selected households and household members (based upon the Framingham model and other cohorts) are of assistance in the design and implementation of new and cost-effective interventions and policies in South Asia [16]–[21]. Accordingly, we chose to study a multi-ethnic, densely populated, impoverished community of 2.5 million located in south-eastern Karachi, adjacent to a major industrial area. The study is based out of a unique high-quality general hospital providing free in-patient care to this area, complemented by community-based health services and a research program operated by a public health team. A small number of NCD prevalence studies in Karachi dating back almost two decades are currently all the data that are available at the population level in Pakistan including urban Karachi. These data are now considerably outdated, and do not account for recent social, economic and behavioral changes that have taken place, especially over the past decade [22]–[24].

The present study was designed as a cross-sectional baseline survey to establish the basis of a unique ‘Framingham-like’ Pakistan cohort with the objective of measuring the prevalence and risk factors for hypertension, obesity, diabetes, coronary artery disease and hepatitis B and C infection in a multi-ethnic, middle to low income population of Karachi. In this report we present findings from the first phase of the cross-sectional baseline survey.

## Materials and Methods

### Population and Research Setting

The Indus Hospital is a 150-bed private tertiary care health facility providing high quality care free-of-cost. It is located in Korangi town, to the east of Karachi port, and is surrounded by one of Pakistan’s largest industrial zones. The Hospital consists of a team of local and expatriate professionals who provide specialized medical and surgical care with an emphasis on innovation and research. The hospital’s outpatient department has around 300 general visits and 400 specialty clinic visits each day. A separate facility for tuberculosis caters to another 350 visits a day. Indus Hospital’s direct catchment population (Korangi town, Landhi town and parts of Bin Qasim town) is a multi-ethnic community of approximately 2.5 million comprised of regional and sub-national migrant settlements adjacent to historical fishing villages along the south-eastern Karachi coast. During the 1960s the area developed as a satellite town to house squatter settlement populations and multi-ethnic Muslim refugees (known as Muhajirs) from post-partition India. The catchment population has evolved into a conglomeration of migrant communities seeking employment in the neighboring industrial zone and Karachi’s expanding urban economy [25].This study was based at the Indus Hospital Research Center (IHRC) whose community outreach programs for this population focus on prevention and early detection of disease, encouraging community involvement and ownership.

### Sampling Frame

Between 2006 and 2009 we completed the physical mapping of all residential structures and health care facilities in the catchment population, ascribing unique household identification numbers, and digitizing these maps using a Geographical Information System (GIS). Household selection for the Indus Hospital Community Cohort (IHCC) was performed on these GIS maps.

### Baseline Survey

A baseline survey was designed to measure the prevalence and risk factors for hypertension, obesity, diabetes, coronary artery disease and hepatitis B and C infection. A two-step implementation was planned for the baseline survey in which phase 1 was completed between March 2010 and August 2011, while phase 2 is planned to start in 2013. The phased approached allowed efficient use of funding resources while also generating data and lessons for the second expansion phase. During the first year we developed standard operating procedures for longitudinal studies, data management protocols, and invested in long-term specimen storage facilities.

### Ethics Statement

Protocols, instruments and consent forms for this study were approved by the Institutional Review Board at Interactive Research and Development (IRD). Written informed consent was obtained from all participants prior to collection of survey data or specimens. In the case of minors (participants less than 15 years of age) we obtained written informed consent from a parent.

### Study Design and Sampling Methods

We selected two administrative areas in the Indus Hospital catchment region for baseline enrollment of a random selection of cohort households. A community-based sampling frame was chosen for cohort representativeness. Since detailed socio-economic data is not available for these survey areas, we used our observed differences in location, settlement patterns and known ethnic diversity of these areas in the selection and planning process. The two administrative units selected were based on relative proximity to Indus Hospital and apparent socio-economic diversity. Proximity to Indus Hospital facilitated effective field operations, monitoring and clinical specimen management.

No recent or reliable census data was available for Pakistan for constructing the sampling frame. We developed the sampling frame for the study population using a GIS database consisting of uniquely numbered household structure maps. We enrolled households using a systematic random sampling design by computing the sampling interval *k* as the ratio of population to sample size and then choosing a random starting point in each neighborhood between 1 and *k.* Every 9^th^ household was systematically selected for enrolment in the IHCC cohort demographic survey.

### Community Outreach

Prior to visiting households we recruited local community members as social mobilizers to motivate household members to participate in the study. Social mobilizers used promotional material and engaged in awareness-raising activities including community focus groups, placing banners in community areas and distributing pamphlets in priority neighborhoods to raise awareness regarding the study. They also enlisted the support of local community organizations and community leaders in motivating households to understand the benefits of the study and encourage participation.

### Data Collection

The protocols and instruments used in this study were modeled in part on validated material from a successful cohort developed by our colleagues in an underserved population of Mexican Americans in South Texas [16]. Household demographic questions were based on the Pakistan Demographic and Health Survey (DHS) and the Pakistan Social and Living Standards Measurements (PSLM) survey [26], [27]. Modules on anthropometry were adopted from the WHO STEPS protocol and questions related to tobacco use from the Global Adult Tobacco Survey (GATS) [28]–[30]. Validated protocols and questionnaires were modified for local cultural and social needs and to include additional information about diseases of local importance in the target population. All protocols and instruments were translated into Urdu, and questionnaires were back-translated and validated in the community to be studied. Staff was extensively trained in administration of the questionnaires and quality control procedures were put in place through regular field supervision of interviews and daily review of collected data.

#### Recruitment and staff

We recruited a multilingual team of six field workers and trained them in interview ethics and survey interview techniques and procedures. All household surveys were conducted in locally spoken languages; Urdu or Sindhi. The health examination was conducted on the mobile health unit (MHU), a specially modified medical truck equipped for study requirements and parked in convenient locations for the households being surveyed. The health examination team on the MHU consisted of a physician, two nurses, a hospital trained phlebotomist, an ultrasonographer, an optometrist, an administration support officer and a driver.

#### Household interview survey

After obtaining informed consent from the head of each household, the household interview survey was administered to him or her, or another consenting adult if the head of household was not present. Household information was collected on demographic characteristics of all members, housing structure, household asset ownership and media access behaviors.

Following the household survey, we obtained written informed consent from three randomly selected household members consisting of a child below 15 years of age and two adults from the age groups 15–29 years and 30 years and above. For children below 15 years of age, written informed consent was obtained from a parent. The participants were invited to complete a health examination survey at the MHU at a designated time and location.

#### MHU health examination survey

When participants visited the MHU for their scheduled visit, a health examination questionnaire was administered that included detailed information on personal medical history, family medical history, use of medical screening services and medication, physical activity, tobacco use, diet and vision, and risk factors for NCDs.

#### Clinical examination

Following the questionnaire, we obtained anthropometric measurements (height, weight, waist circumference, and hip circumference) and the average of three seated blood pressure readings 10 minutes apart using standardized instruments (OMRON -4, Omron Corporation, Kyoto, Japan) and WHO protocols [29].

#### Specimen collection

Blood specimens were drawn from all consenting participants (10 mL for adults, 6 mL for children). All specimens were stored in the MHU refrigerator at 4°C for under 4 hours prior to being transported on ice to the Indus Hospital clinical laboratory for appropriate processing, aliquoting, testing and banking.

Upon completion of the interview and provision of specimens, the participants were provided the rupee equivalent of $0.80–1.00 as well as Indus Hospital monogrammed towels, biscuits, and baby products to acknowledge their time and effort.

### Specimen Processing and Storage

Plasma glucose was measured using a glucose oxidase method (RANDOX GODPAP, UK), and glycated hemoglobin (A1c) through a boronate affinity assay using a Nycocard reader (Axis-Shield, Norway). Lipids were measured using the Randox cholesterol kit (RANDOX Cholesterol (CHOL), UK). Hepatitis B and C infections were confirmed using a chemiluminescence IgG antibody assay (Johnson & Johnson, VITROS ECi). The remainder of each blood specimen used for glucose testing was separated into component phases and stored permanently at −80°C at the Indus Hospital.

### Data Management and Confidentiality

All study questionnaires were administered as pre-coded paper forms. Completed forms were checked for errors by supervisors and then double entered into a Microsoft Access 2007 data entry program developed by the data management team at IRD. Entered forms containing identifiers were stored in cabinets behind double locked doors and identifiers were delinked from study databases to maintain confidentiality.

### Definitions Used

Literacy was defined as the respondent’s own description of their ability to read, to write one page in any language and to perform simple arithmetical calculations. Improved sanitation facility was defined as one that hygienically separates human excreta from human contact and improved drinking-water source was defined as one that, by nature of its construction or through active intervention, is protected from outside contamination [31]. Any form of tobacco use was considered as an NCD risk factor, with a distinction made for the type of tobacco used. Underweight was defined as BMI below 18.5 kg/m^2^, normal weight as BMI from 18.5 kg/m^2^ to below 25.0 kg/m^2^, overweight as BMI between 25.0 kg/m^2^ and less than 30.0 kg/m^2^ and obesity as BMI ≥30 kg/m [6]. Abdominal obesity was defined as a waist circumference of ≥90 cm in men and ≥80 cm in women [32]. Pre-hypertension was defined as a systolic blood pressure of 120–139 mmHg or diastolic blood pressure between 80–89 mmHg, and hypertension was defined as systolic blood pressure ≥140 mmHg, or a diastolic blood pressure of ≥90 mmHg [33]. Individuals with A1c measurement between 5.5% and below 6.5% were considered to have ‘pre-diabetes’ while those with A1c≥6.5% were considered to have diabetes mellitus [34]. A borderline high serum cholesterol level was defined as total cholesterol between 200–239 mg/dl. Hypercholesterolaemia was defined as total cholesterol ≥240 mg/dl [35].

### Statistical Methods

De-identified data were analyzed using Stata SE 11.1 (StataCorp LP, College Station, Texas) and SPSS version 19 (SPSS Inc, Chicago, IL). We calculated mean values for body mass index (BMI), waist circumference, blood pressure and biochemical variables. We report the prevalence and 95% confidence intervals of all major risk factors for NCDs surveyed in this study.

## Results

Eight hundred twenty-two households were approached in the first phase of the study between March 2010 and August 2011. Six hundred sixty seven (82%) of the households approached enrolled in the IHCC study. From the enrolled households, 1778 household members from the three age groups were randomly selected for the detailed health examination and 1508 (85%) consented to participate. In phase one, reported here, we completed detailed health examination and specimen collection on 780 participants and achieved a participation rate of 52% for randomized individuals. Nearly all (99%) of the 780 completed the health examination survey, and anthropometry was recorded on 98%. Blood specimens were provided by 87% (75% in children less than 15 years, 93% in adults 15 years or greater). In most cases when blood specimens were not collected, the participant had refused.

The baseline characteristics of cohort household members and selected participants are summarized (Table 1). There were a total of 4,825 household members enrolled from 667 cohort households. This represented a mean household size of 7.2 persons (SD 3.4) with 90% male heads of household and a mean household crowding index of 4.2 persons per sleeping room (SD 2.0). The highest proportion of household members (41%) was aged 14 years and younger followed by those aged between 15–29 years (32%). The major ethnicities in our sample were Sindhi (38%), Bengali (23%) and Muhajir (10%). The majority of the population over 5 years of age had not received any schooling (58%) and only one-third were literate (33%). 10% of the household members of employable age (older than 10 years) were economically inactive [27].

**Table 1:** Socio-demographic characteristics of household members and selected participants.

*Characteristic category*	*Sub-category*	*Household members N = 4825*		*Enrolled participants N = 780*		*p-value* a
		n	%	n	%	
Age (years)b,c	0–14	1,964	41	284	36	
	15–29	1,549	32	231	30	
	30–44	684	14	147	19	
	45–59	464	10	88	11	
	60–74	133	3	25	3	
	75+	29	1	4	<1	
Sex	Males	2,469	51	334	43	<0.01
	Females	2,356	49	446	57	
Education level^d^	No schooling	2,375	58	387	57	0.66
	Any schooling	1,755	42	295	43	
Literacy^d^	Not literate	2,852	67	459	66	0.35
	Literate (reading, writingand numeracy)	1,375	33	237	34	
Employment Status^e^	Currently Employed	1,360	90	168	89	0.94
	Unemployed	159	10	20	11	

a p-value for X^2^ test for homogeneity of proportions by the specific sub-categories of each characteristic. If p<0.05, the characteristic proportion distribution (e.g. sex) is different between household members and enrolled participants.

b X^2^ test not performed for age since participants were selected based on their age so the distribution is not expected to be similar to that of all household members.

c Age was missing for two household members.

d Education level and literacy was obtained for individuals aged ≥5 years only.

e Employment status was obtained for individuals of employable age (≥10 years) only.

Table 1 also shows the same baseline characteristics for the 780 participants on whom we completed the detailed health examination. The distribution of education, literacy and employment characteristics were very similar to that of household members with no significant differences (p>0.05), suggesting that these participants were representative of the population from which they were selected. However, a greater proportion of females (57% vs. 49% in households) health examinations were completed and participants were 1.49 times more likely to be female (p<0.01).

Table 2 presents the lifestyle characteristics of the 667 households that participated in the household survey. The majority of households (83%) owned the homes they were living in, and most of the homes were semi-permanent structures, with 85% having cemented floors. However, only 21% of the households had reinforced cement concrete roofs. Most households (98%) used an improved sanitation facility, while only 68% percent of the households had access to an improved drinking-water source. A similar proportion of households had access to electricity (76%) or natural gas (78%). Mobile phone and cable television were the two primary communication technologies owned by a high proportion of households, 83% and 69% respectively, while computer access was insignificant (4%) in this population. Access to private transportation was low and only 21% of households owned any kind of motorized vehicle.

**Table 2:** Household and lifestyle characteristics.

*Characteristic category*	*Sub-category*	*Number of households (N = 667)*	
		*n*	*%*
Living environment	Self-owned home	556	83
	Cement floor	568	85
	Cement block wall	627	94
	Reinforced concrete cement roof	139	21
Access to utilities	Improved sanitation facility	654	98
	Natural gas	521	78
	Electricity	509	76
	Improved drinking-water source	455	68
Ownership of kitchen appliances	Stove^a^	550	91
	Refrigerator	140	21
	Cooking range	60	9
	Microwave	8	1
Ownership of or access to communication technology	Mobile phone	553	83
	Cable television	459	69
	Computer	30	4
Transportation	Motorcycle	129	19
	Bicycle	42	6
	Motor vehicle	16	2

a The question regarding ownership of a stove was added at a later date, therefore responses were only available for 603 households (550/603 = 91%).

The mean and median values, as well as inter-quartile range (IQR) of BMI, waist circumference, blood pressure, glycated hemoglobin and total cholesterol among adults aged 15 years and older are summarized in Table 3, and the prevalence of selected anthropometric, biochemical and behavioral risk factors are presented in Table 4. Our study identified 20% of the adult population as overweight and 8% obese with a mean BMI of 22.4 kg/m^2^. More than half (53%) of all females had abdominal obesity – twice the proportion of males with the same risk factor (27%). At the same time almost a quarter of the participants (23%) were underweight. In our sample, 18% of the adult participants were hypertensive, 8% were diabetic and 4% had high cholesterol. An additional 39%, 40% and 12% had pre-hypertension, pre-diabetes and borderline high cholesterol, respectively. We also identified a high use of smokeless tobacco (45%) compared to smoking tobacco (12%) among the participants. Nearly a quarter (24%) of participants of all ages had a previous hepatitis B virus infection (HBV), while 8% of participants had been exposed to hepatitis C virus (8%). Over 8% of the participants had received greater than three therapeutic injections in the preceding 12 months, a known risk factor of hepatitis C virus (HCV) transmission in Pakistan.

**Table 3:** Table **3**. Mean, median and IQR of NCD risk factors^a^.

*Risk factor*	*Mean (SD)*	*Median*	*IQR*
BMI (kg/m^2^)	22.4 (5.1)	21.5	18.6–25.9
Waist circumference (Males, cm)	82.4 (15.0)	79.8	72.0–91.0
Waist circumference (Females, cm)	81.6 (13.7)	80.0	71.0–92.0
Systolic blood pressure (mmHg)	122.9 (18.1)	120.2	111.0–130.0
Diastolic blood pressure (mmHg)	76.9 (11.3)	75.7	69.7–83.7
Glycated hemoglobin (A1c) (%)	5.5 (1.0)	5.4	4.9–5.8
Total cholesterol (mg/dL)	163.6 (42.0)	158.0	137.0–186.0

a *Age ≥15 yrs unless otherwise specified.*

**Table 4:** Prevalence of risk factors for NCD and infectious diseases^a^.

*Risk factor category*	*Sub-category*	*Prevalence n (%)*	*95% CI*
Anthropometric factors	Underweight (BMI<18.5 kg/m^2^)	117 (23)	20–27
	Normal weight (18.5≤BMI<25.0 kg/m^2^)	241 (48)	44–53
	Overweight (25.0≤BMI<30.0 kg/m^2^)	102 (20)	17–24
	Obese (BMI≥30.0 kg/m^2^)	39 (8)	5–10
	Abdominal obesity (Males, waist circumference≥90 cm)	50 (27)	21–34
	Abdominal obesity (Females, waist circumference≥80 cm)	169 (53)	48–59
	Prehypertension (Systolic: 120–139 mmHg, Diastolic: 80–89 mmHg)	195 (39)	35–43
	Hypertension (Systolic: ≥140 mmHg, Diastolic: ≥90 mmHg)	90 (18)	15–21
Biological factors	Pre-diabetes (A1c: 5.5–6.4%)	185 (40)	35–44
	Diabetes (A1c: ≥6.5%)	37 (8)	5–10
	Borderline high cholesterol (Total cholesterol: 200–239 mg/dl)	55 (12)	9–14
	Hypercholesterolaemia (Total cholesterol: ≥240 mg/dl)	21 (4)	3–6
	Hepatitis B (all ages)	153 (24)	20–27
	Hepatitis C (all ages)	53 (8)	6–10
Behavioral factors	Smoking tobacco users	56 (12)	9–16
	Smokeless tobacco users	203 (45)	41–50
	Frequent use of therapeutic injections (>3 in 12 months)	65 (8)	6–10

a *Age ≥15 yrs unless otherwise specified.*

## Discussion

We report baseline cross sectional data from a randomly recruited cohort in a typical transitional community in Karachi. Our results show high rates of anthropometric and biological markers of adverse health in this community. Mean (22.4 kg/m^2^) and median BMI (21.5 kg/m^2^) were comparable to a population-based national estimate of average BMI for adult Pakistanis, as well as in a more recent community-based study from India [36], [37]. Mean BMI was comparable to that in other Asian populations as well [38]. Greater than one in four participants was overweight or obese. This prevalence was higher than that found among urban residents from the National Health Survey of Pakistan (NHSP) 1990–94, despite the inclusion of younger adults between the ages of 15 and 24 in our sample [39]–[41]. Meanwhile, the estimate for overweight and obesity was found to be similar to other South Asian populations, and much higher than in other Asian settings such as urban Indonesia and Vietnam [37], [42], [43]. A similar pattern was observed with waist circumference and abdominal obesity, with comparable average estimates and prevalence as in other Pakistani and South Asian populations [37], [41], [44]. The overall prevalence of hypertension in adults aged 15 years and above was 18%. This prevalence was lower than national estimates on urban Pakistanis from the last National Health Survey of Pakistan 1998 [45]. However, another nationally representative study looking at ethnic differences identified similar low rates among Sindhis [22]. This ethnicity represents a majority of the IHCC cohort population, potentially explaining the overall lower-than-national prevalence of hypertension. These rates were also lower compared to other Asian populations [37], [43]. Another third of the population were pre-hypertensive and hence at elevated risk for the condition.

Our study was the first in Pakistan to measure glycated hemoglobin levels in randomly selected participants from the general population. Most previous studies comparing A1c levels have been carried out on diabetic or other ambulatory patients in the Pakistani hospital setting [46], [47]. Compared to a survey conducted among the adult English population, the mean A1c level found in our sample was lower than that found in South Asian immigrants (5.5% vs. 5.9%, respectively) [48]. However, this difference may be partially attributed to the inclusion of younger adults aged less than 35 years in our sample who may have lower circulating glycated hemoglobin. Moreover, our study identified eight percent of the population with diabetes, and this level was higher than that identified previously in the National Diabetes Survey 1994 using 2-hour venous plasma glucose [49]. More importantly, we found that an additional two in five individuals had A1c levels indicative of impaired fasting glucose or glucose tolerance. In addition only 3.7% (95% CI: 2.4–5.9) of all individuals screened for diabetes mellitus were on treatment and most of these patients (59%) were still hyperglycemic. Average total cholesterol (163.6 mg/dl) in our population was found to be similar to that in Pakistani ambulatory patients without heart disease, but lower than that found in other South Asian general populations [37], [44]. Compared to data from the NHSP 1990–94 where 13% of Pakistani adults had elevated serum cholesterol our study found a slightly higher percentage of adults with borderline-to-high cholesterol (16%), despite the inclusion of younger adults in our sample [39]. This proportion was similar to that found in other urban South Asian settings [37].

The prevalence of current daily smoking (12%) was found to be lower in our population than previously reported for urban areas in Pakistan (15%), the difference likely owing to the inclusion of adults aged below 18 years in our sample as compared to that used by WHO [50]. More alarming was the high level of smokeless tobacco use (45%) in this population. This finding was similar to previous studies in adult patients at family clinics (52%), and a squatter settlement in Karachi (nearly 40%) [51], [52].

These findings suggest a high burden of non-communicable diseases maybe emerging in developing megacities like Karachi. As we follow this cohort over time, we anticipate that we will see the burden increase. The combined burden of NCD and infectious diseases on Karachi is very likely undermining economic growth, resulting in new patterns of health inequities. We recently reported very high rates of tuberculosis (350 per 100,000 population) in an area that included the communities surveyed here [53]. Along with the 8% of the general population infected with HCV in this cohort, these infectious diseases and others will continue to take an unacceptable toll on lives, health expenditures and economic productivity well into the future. While a young population with increasing life expectancy rates and declining infant mortality signifies a demographic transition in Karachi, this is characterized by rapid and unplanned urbanization leading to increased social inequality in the form of low education attainment, limited access to safe drinking water, substandard housing infrastructure and crowded living conditions. Meanwhile, the public health care system has not been able to cope with rapid population growth and changes in disease patterns [26], [54].

Our results concur with emerging studies in major urban centers of South Asia identifying the linkages between levels of urbanicity and lifestyles characterized by unhealthy nutrition, reduced physical activity and tobacco consumption [55]–[59]. Recent studies in major urban centers of India and Sri Lanka developed an urbanicity scale to measure the linkages between urban environment and NCD risk factors. Both studies concluded that a relationship exists between urbanicity and NCD risk factors, where urbanicity represented access to markets, transport, communication, health care services, educational attainment and population density [56], [57].

There are few comprehensive studies from developing countries on NCD risk factors in communities such as this one, and limited experience in the setting up of NCD and infectious disease surveillance models in low-resource urban settings. Ours is the first such study among randomly selected households from a transition population in Karachi, and it required the investment of considerable up front time and resources in participatory planning and community mobilization. This outreach effort proved to be valuable in enlisting the support of community organizations and leaders while building confidence in the Indus Hospital in whose catchment area they reside. The community engagement strategies we have developed will be invaluable in our next enrollment phase and future cohort follow up. We anticipate that our investment in the long-term community relationship will help us in designing socially acceptable and community-based participatory interventions in the future. Our findings reinforce the need to develop methodologies and tools to monitor NCD risk factors in developing countries that may help advance community-based intervention models to prevent NCDs in transition communities.

There were several limitations to our study. First, our findings are limited to baseline data. Longitudinal follow up of the cohort is needed to demonstrate longer term health outcomes. We plan to generate further data through the expansion of enrollment in phase two and follow up of the current cohort to yield a more representative sample for Karachi. Another limitation was low participation and underrepresentation of men in the health examination at the mobile health unit despite a significantly higher number of informed consents attained at the household. In the local culture, permission of the head of household was required for participation in the health examination. The household head was usually a male and was absent for extended periods for work. Among actual attendees we found more women than men because they were often at home. Proliferation of non-profit, governmental, commercial and academic research in poor and marginalized communities may have lowered the interest and motivation for participation in some households. Similar patterns of declining participation have been observed in the other parts of the world including among major epidemiological studies in developed countries [60]. Known diabetes was not taken into account when estimating the prevalence of diabetes in this study as access to diabetes care in these populations is limited and individuals with diabetes likely suffer from persistent hyperglycemia. The missing data for study was less than 1% for most variables and not more than 2.5%. Missing value analysis showed that the data were missing completely at random and hence this is only a minor limitation.

The strengths of the study are significant, as we recruited the first randomly selected cohort in Karachi to generate data on the burden of NCD and infectious diseases and prevalence of associated risk factors. Accurate sampling of households in low- and middle-income megacities is challenging. The absence of recent census data necessitated the labor-intensive creation of a GIS household database allowing efficient and accurate sampling. The representativeness and success of random selection is suggested by the near-identical distribution of household member age and gender distribution to that found by the Pakistan Demographic and Health Survey 2006–07 [26].

Our findings indicate that the future burden of disease – infectious and non-communicable - will likely increase in a population with already poor access to quality health care. Karachi is one of the fastest growing urban centers in the world, as suggested by preliminary 2011 census data [54]. The social, economic and health implications of this rapid growth will be long-lasting, and there will be a great need for community-based data to plan for health promotion in these communities. Public health programming in Pakistan has been characterized by a range of vertical disease-specific programs that include the national programs for immunization, nutrition, maternal child health and programs aimed at prevention and control of malaria, tuberculosis and HIV/AIDS. The national health system has paid little attention to controlling NCDs and developing an integrated NCD surveillance model. This represents a major weakness in Pakistan’s public health planning in particular for its rapidly expanding multi-ethnic urban communities [61]. Recent changes brought about by a hasty abolition of the Ministry of Health and devolution of its responsibilities to the provinces increased challenges for local public health systems, but also provide an opportunity for a new beginning [62]. Our findings highlight the need for developing NCD intervention programs in coordination with existing disease control programs, and for establishing active surveillance for effective planning, implementation, and evaluation. Comprehensive control of NCD risk factors and infectious diseases in multi-ethnic Asian megacities has become an absolute necessity.
